# The association of blood metals with latent tuberculosis infection among adults and adolescents

**DOI:** 10.3389/fnut.2023.1259902

**Published:** 2023-11-03

**Authors:** Jinyi Wu, Kai Wang, Fengxi Tao, Qingwen Li, Xin Luo, Fang Xia

**Affiliations:** Department of Public Health, Wuhan Fourth Hospital, Wuhan, China

**Keywords:** blood metals, latent tuberculosis infection, NHANES, LTBI, TB

## Abstract

**Objective:**

We aimed to investigate the relationship of metal exposure and latent tuberculosis infection (LTBI) among US adults and adolescents.

**Methods:**

Participants from the National Health and Nutrition Examination Surveys (NHANES 2011 ~ 2012) were included. Multiple logistic regression models were used to explore the associations between metal exposure and LTBI. A total of 5,248 adults and 1,860 adolescents were included in the present analysis.

**Results:**

For adults, we only found a positive association between total mercury and LTBI (OR: 1.411; 95% CI: 1.164 ~ 1.710) when used as a continuous variable. Compared with Q1, Q4 increased the prevalence of LTBI (2.303; 1.455 ~ 3.644) when used as a quartile. The OR of total mercury and LTBI was higher among females (1.517; 1.009 ~ 2.279), individuals aged 45 ~ 64 (1.457; 1.060 ~ 2.002), and non-Hispanic White individuals (1.773; 1.316 ~ 2.388). A relationship was observed among only participants with obesity (1.553; 1.040 ~ 2.319) or underweight (1.380; 1.076 ~ 1.771), with college or above (1.645; 1.184 ~ 2.286), with PIR > 3.0 (1.701; 1.217 ~ 2.376), reported smoking (1.535; 1.235 ~ 1.907) and drinking (1.464; 1.232 ~ 1.739). For adolescents, blood manganese was positively associated with LTBI. The OR and 95% CIs for each one-unit increase in the log-transformed level of blood manganese with LTBI were 9.954 (1.389 ~ 71.344).

**Conclusion:**

Significant associations were observed in girls, aged ≥12 years and in the non-Hispanic white population. In conclusion, total mercury is associated with an increased prevalence of LTBI among adults and positive association between blood manganese and LTBI was observed among adolescents. Further studies should be conducted to verify the results and explore potential biological mechanisms.

## Introduction

In 2020, approximately 1.5 million people died from tuberculosis (TB), and it was estimated that 10 million people fell ill with tuberculosis (TB) worldwide. TB was the 13th leading cause of death and the second leading infectious killer after COVID-19. By 2022, a total of 13 billion dollars are needed annually for TB prevention, diagnosis, treatment and care in the US (1). Fortunately, TB is curable and preventable. According to the United Nations High Level Meeting on TB in 2018, effective management and treatment of latent tuberculosis infection (LTBI) is one of the TB preventive care approaches to control and reduce the incidence of newly diagnosed active TB (2). Individuals with LTBI are clinically asymptomatic; however, to diagnose and to treat them for LTBI could be beneficial in preventing TB (3). A previous study revealed that major reductions in US tuberculosis incidence could be achieved if LTBI treatment was substantially increased (4), and LTBI was considered as the final frontier of tuberculosis elimination.

LTBI is an asymptomatic status in which individuals demonstrate an immunological response to mycobacterium tuberculosis that confers a heightened risk of subsequently developing TB (5), and approximately one-third of the world’s population may harbor LTBI. LTBI is caused by mycobacterium tuberculosis; however, many other factors (6), such as socioeconomic status (7) and diabetes (8), also increase the risk of LTBI. Previous studies showed that heavy metals were associated with adverse impacts on immune function and lung host defense (9, 10), which may increase the risk of LTBI. It was reported that an exposure-response relationship between cumulative respirable metal dust exposures with deterioration of lung function among steel workers. The economic cost to society from COPD is tied to dysfunctional innate immune responses to infection, leading to repetitive and severe exacerbations of this existing lung disease (9, 10). In addition, some studies also indicated that chronic low-grade inflammation may contribute to susceptibility to TB infection (11), while metals such as zinc, lead and cadmium have been demonstrated to be associated with systemic inflammation (12, 13). Therefore, we speculate that metals may be associated with LTBI.

To address the gap in knowledge related to metal exposure and LTBI, we conducted the study to determine the association between blood metals including lead, cadmium, total mercury, selenium and manganese and LTBI using the National Health and Nutrition Examination Survey (NHANES).

## Methods

### Study population

The study population was from NHANES 2011 ~ 2012 and data were obtained by questionnaire and interview, mobile physical examination and laboratory tests with a complex, multistage, probability sampling method. Details of NHANES have been described online^1^ (14–17). In the present analysis, participants with basic characteristics (*N* = 9,756), blood metals (*N* = 8,956), and LTBI (*N* = 7,821) were first enrolled. After excluding data without incomplete information on blood metals (*N* = 1,810) and LTBI (*N* = 669), a total of 7,108 individuals (*N* = 5,248 for adults and *N* = 1,860 for adolescents aged 12–17 years old) were included. The flowchart of the study is presented in Supplementary Figure S1. The NHANES protocol was approved by the National Center for Health Statistics Institutional Review Board, and written informed consent is obtained.

### Definition of LTBI

QuantiFERON-TB gold In-Tube (QFT-GIT) was analyzed according to manufacturer instructions. The results were interpreted according to guidelines from the Centers for Disease Control and Prevention (CDC) for using interferon-gamma release assays (IGRAs) (18). Participants with positive QFT-GIT results were classified as LTBI positive, and participants with negative QFT-GIT results were classified as LTBI negative. Participants with indeterminate QFTGIT results or missing were classified as LTBI positive if induration ≥10 mm in response to PPD regardless of participants’ LTBI risk factors (19, 20).

### Measurement of blood metals

We choose the blood metals according to data from NHANES (15–17). Whole blood specimens are processed, stored, and shipped to the Division of Laboratory Sciences, National Center for Environmental Health, and Centers for Disease Control and Prevention for analysis. The metals measured in whole blood were lead, cadmium, total mercury, selenium and manganese. The detection limit for all analytes was constant in the data set. The lower detection limits for lead were 0.25 μg/dL, and 0.16 μg/L for cadmium, 30 μg/dL for selenium, 1.06 μg/dL for manganese, and 0.16 μg/L for total mercury. Among the samples where values were below the limit of detection, they were imputed as the lowest LOD value for each metal.^2^

### Ascertainment of covariates

We included covariates based on previous reports (15–17). Gender, age, race/ethnicity (Mexican American, non-Hispanic white, non-Hispanic black, non-Hispanic Asian and others), educational level (less than high school, high school or equivalent, and college or above), family income-poverty ratio (PIR), smoking status (only for adults), drinking status (only for adults), and body mass index (BMI) were obtained by interviews and physical examinations. BMI was calculated as weight (kg) divided by the square of height (m^2^), and was categorized into three groups: <25, 25 ~ 30 and ≥ 30 kg/ m^2^. PIR was grouped into three categories: 0–1.0, 1.1–3.0, and > 3.0. Current smokers were defined as those who smoked at least 100 cigarettes and smoked at the time of the survey (only for adults). Current drinkers were defined as those who had at least 12 alcohol drinks per year (only for adults).

### Statistical analysis

Complex survey design factors including sample weights, clustering, and stratification were considered for all analyses with instructions for using NHANES data. We compared baseline characteristics by LTBI in the two intervals by using the Rao-Scott *χ^2^* test for categorical variables and analysis of variance and Kruskal-Wallis test adjusted for sampling weights for continuous variables. Logistic regression models were used to estimate the odds ratios (ORs) with 95% confidence intervals (CIs) for each metal and LTBI. The baseline age (years, continuous), gender, race/ethnicity (non-Hispanic white, non-Hispanic black, Mexican American, and others) and education level (less than high school, high school or equivalent, and college or above) were adjusted in model 2. Furthermore, family income-poverty ratio level (0–1.0, 1.1–3.0, >3.0), BMI (<25, 25 ~ 30 and ≥ 30 kg/ m^2^), smoking status (no, yes) and drinking status (yes, no) were adjusted in model 3. In subgroup analyses, we examined the bivariate association between all participant characteristics and lifestyles among adults and that between gender, age and PIR among adolescents adjusted for gender, age, race, education, income, BMI, smoking status and drinking status.

All statistical analyses were performed using SAS version 9.4 (SAS Institute Inc., Cary, NC, United States) with a 2-sided *p* < 0 0.05 considered statistically significant.

## Results

### Basic characteristics

Tables 1 and 2 show the basic characteristics of the 5,248 adults and 1,860 adolescents. For adults, 2,599 (49.52%) were males, and more than 50% were individuals with college and above educational levels. The weighted genome means of metals (95%CI) including lead, cadmium, total mercury, selenium, and manganese were 1.067 (1.003 ~ 1.136), 0.332 (0.317 ~ 0.347), 0.849 (0.742 ~ 0.972), 192.908 (190.047 ~ 195.812), and 9.101 (8.954 ~ 9.250) μg/dL, respectively. For adolescents, 951 (51.13%) were boys, 1,019 (54.78%) were adolescents aged 6 ~ 12 years old. The weighted genome means of metals (95%CI) including lead, cadmium, total mercury, selenium, and manganese were 0.604 (0.564 ~ 0.647), 0.146 (0.141 ~ 0.151), 0.355 (0.304 ~ 0.414), 182.502 (177.846 ~ 187.281), and 10.226 (10.002 ~ 10.456) μg/dL, respectively. The distribution of blood metals in US adults and adolescents are presented in Supplementary Tables S1, S2, and significant differences were observed for all metals between most groups.

**Table 1:** Basic characteristics of the adults in NHANES 2011–2012 according to LTBI (*N* = 5,248).

Characteristics	All participants	Non-LTBI (4742)	LTBI (506)	Rao-Scott *χ*^2^	*p* value
**Gender**				11.0154	0.0009*
Male	2,599 (49.52)	2,306 (48.63)	293 (57.91)		
Female	2,649 (50.48)	2,436 (51.37)	213 (42.09)		
**Age**				23.2327	<0.0001*
18 ~ 44	2,461 (46.89)	2,338 (49.30)	123 (24.31)		
45 ~ 64	1713 (32.64)	1,490 (31.42)	223 (44.07)		
≥65	1,074 (20.46)	914 (19.27)	160 (31.62)		
**Race**				225.424	<0.0001*
Mexican American	538 (10.25)	462 (9.74)	76 (15.02)		
Non-Hispanic White	1926 (36.70)	1854 (39.10)	72 (14.23)		
Non-Hispanic Black	1,372 (26.14)	1,250 (26.36)	122 (24.11)		
Non-Hispanic Asian	712 (13.57)	574 (12.10)	138 (27.27)		
Others	700 (13.34)	602 (12.70)	98 (19.37)		
**Educational levels**				41.1142	<0.0001*
Less than high school	1,251 (23.84)	1,059 (22.33)	192 (37.94)		
High school or equivalent	1,122 (21.38)	1,017 (21.45)	105 (20.75)		
College or above	2,875 (54.78)	2,666 (56.22)	209 (41.30)		
^**a**^ **PIR**				14.9649	0.0006*
0–1.0	1,272 (26.44)	1,139 (26.09)	133 (29.89)		
1.1–3.0	1862 (38.71)	1,680 (38.49)	182 (40.90)		
>3.0	1,676 (34.84)	1,546 (35.42)	130 (29.21)		
^**b**^ **BMI, kg/m** ^**2**^				0.5966	0.7421
<25	1,699 (32.86)	1,533 (32.83)	166 (33.07)		
25–30	1,639 (31.70)	1,468 (31.44)	171 (34.06)		
≥30	1833 (35.45)	1,668 (35.72)	165 (32.87)		
^**c**^ **Smoking status**				3.067	0.0799
No	2,835 (57.09)	2,573 (57.60)	262 (52.51)		
Yes	2,131 (42.91)	1894 (42.40)	237 (47.49)		
^**d**^ **Drinking status**				11.9029	0.0006*
No	3,419 (72.59)	1,142 (26.71)	149 (34.33)		
Yes	1,291 (27.41)	3,134 (73.29)	285 (65.67)		
**Blood metals (ug/dL) (GM, 95%CI)**					
Blood lead	1.067 (1.003 ~ 1.136)	1.051 (0.989 ~ 1.118)	1.374 (1.210 ~ 1.560)	−1.9	0.075
Blood cadmium	0.332 (0.317 ~ 0.347)	0.329 (0.314 ~ 0.345)	0.382 (0.337 ~ 0.432)	−0.44	0.6681
Total mercury	0.849 (0.742 ~ 0.972)	0.832 (0.727 ~ 0.951)	1.205 (0.988 ~ 1.469)	−3.94	0.0011*
Blood Selenium	192.908 (190.047 ~ 195.812)	192.962 (190.038 ~ 195.932)	192.009 (187.698 ~ 196.420)	0.47	0.642
Blood manganese	9.101 (8.954 ~ 9.250)	9.076 (8.935 ~ 9.219)	9.529 (9.027 ~ 10.058)	−1.93	0.0708

LTBI: latent tuberculosis infection; PIR: family income-poverty ratio; BMI: body mass index. ^a^438 individuals missing; ^b^77 individuals missing; ^c^ 282 individuals missing; ^d^538 individuals missing. * *p* < 0.05.

**Table 2:** Basic characteristics of the adolescents in NHANES 2011–2012 according to LTBI (*N* = 1860).

Characteristics	All participants	Non-LTBI (4742)	LTBI (506)	*Rao-Scott* χ^2^	*p* value
**Gender**				0.0271	0.8692
Boy	951 (51.13)	939 (51.09)	12 (54.55)		
Girl	909 (48.87)	899 (48.91)	10 (45.45)		
**Age**				0.8378	0.36
6 ~ 12	1,019 (54.78)	1,010 (54.95)	9 (40.91)		
≥12	841 (45.22)	828 (45.05)	13 (59.09)		
**Race**				3.2549	0.5161
Mexican American	369 (19.84)	364 (19.80)	5 (22.73)		
Non-Hispanic White	422 (22.69)	418 (22.74)	4 (18.18)		
Non-Hispanic Black	557 (29.95)	554 (30.14)	3 (13.64)		
Non-Hispanic Asian	212 (11.40)	207 (11.26)	5 (22.73)		
Others	300 (16.13)	295 (16.05)	5 (22.73)		
^**a**^ **PIR**				3.4157	0.1813
0–1.0	611 (35.07)	605 (35.17)	6 (27.27)		
1.1–3.0	689 (39.55)	678 (39.42)	11 (50.00)		
>3.0	442 (25.37)	437 (25.41)	5 (22.73)		
**Blood metals (ug/dL) (GM, 95%CI)**					
Blood lead	0.604 (0.564 ~ 0.647)	0.605 (0.565 ~ 0.647)	0.546 (0.344 ~ 0.868)	0.37	0.7194
Blood cadmium	0.146 (0.141 ~ 0.151)	0.146 (0.141 ~ 0.151)	0.146 (0.115 ~ 0.186)	0.05	0.959
Total mercury	0.355 (0.304 ~ 0.414)	0.354 (0.302 ~ 0.415)	0.448 (0.343 ~ 0.585)	−0.76	0.4557
Blood Selenium	182.502 (177.846 ~ 187.281)	182.435 (177.767 ~ 187.226)	188.484 (177.564 ~ 200.076)	−1.07	0.2986
Blood manganese	10.226 (10.002 ~ 10.456)	10.203 (9.964 ~ 10.447)	12.529 (10.910 ~ 14.388)	−2.57	0.0198*

LTBI: latent tuberculosis infection; PIR: family income-poverty ratio; BMI: body mass index. ^**a**^118 individuals missing. * *p* < 0.05.

### Associations between blood metals and LTBI among adults and adolescents

The associations between blood metals and LTBI among adults are presented in Table 3. A significant positive association of total mercury with LTBI was found, and OR (95% CI) for each 1-unit increase in log-transformed levels of total mercury with LTBI was 1.411 (1.164 ~ 1.710) after adjusting for all variables. Compared with Q1, Q4 was significantly associated with an increased prevalence of LTBI (2.303; 1.455 ~ 3.644). Furthermore, subgroup analyses of the association of total mercury with LTBI were conducted (Supplementary Table S3). The OR of total mercury and LTBI was higher among females (1.517; 1.009 ~ 2.279), individuals aged 45 ~ 64 (1.457; 1.060 ~ 2.002), and non-Hispanic White individuals (1.773; 1.316 ~ 2.388). In addition, a positive relationship was observed among participants with obesity (1.553; 1.040 ~ 2.319) and underweight (1.380; 1.076 ~ 1.771), with college or above (1.645; 1.184 ~ 2.286), with PIR > 3.0 (1.701; 1.217 ~ 2.376), reported smoking (1.535; 1.235 ~ 1.907), and drinking (1.464; 1.232 ~ 1.739).

**Table 3:** Associations between blood metals and LTBI among adults.

	Model 1	Model 2	Model 3
Blood lead (ug/dl)			
Continuous log-transformed	1.657 (1.398 ~ 1.962)*	1.213 (0.973 ~ 1.513)	1.201 (0.881 ~ 1.639)
Q1 (0.18 ~ 0.68)	Ref.		
Q2 (0.69 ~ 1.08)	2.114 (1.438 ~ 3.109)	1.537 (0.998 ~ 2.367)	1.661 (1.013 ~ 2.723)
Q3 (1.09 ~ 1.73)	2.625 (1.663 ~ 4.143)	1.512 (0.958 ~ 2.388)	1.449 (0.932 ~ 2.251)
Q4 (1.74 ~ 61.29)	3.121 (2.045 ~ 4.763)	1.580 (0.952 ~ 2.623)	1.529 (0.844 ~ 2.770)
Blood cadmium (ug/dl)			
Continuous log-transformed	1.218 (1.023 ~ 1.450) *	1.058 (0.890 ~ 1.259)	0.914 (0.738 ~ 1.132)
Q1 (0.11 ~ 0.20)	Ref.		
Q2 (0.21 ~ 0.33)	1.038 (0.695 ~ 1.551)	0.824 (0.567 ~ 1.198)	0.799 (0.503 ~ 1.270)
Q3 (0.34 ~ 0.61)	1.527 (0.941 ~ 2.476)	1.009 (0.607 ~ 1.676)	0.718 (0.385 ~ 1.336)
Q4 (0.62 ~ 9.30)	1.751 (1.188 ~ 2.581)	1.174 (0.806 ~ 1.709)	0.876 (0.544 ~ 1.413)
Total mercury (ug/dl)			
Continuous log-transformed	1.403 (1.232 ~ 1.598) *	1.282 (1.126 ~ 1.459) *	1.411 (1.164 ~ 1.710) *
Q1 (0.11 ~ 0.42)	Ref.		
Q2 (0.43 ~ 0.83)	1.196 (0.763 ~ 1.875)	1.142 (0.762 ~ 1.710)	1.270 (0.756 ~ 2.135)
Q3 (0.84 ~ 1.86)	1.214 (0.788 ~ 1.870)	1.176 (0.843 ~ 1.642)	1.414 (0.972 ~ 2.059)
Q4 (1.87 ~ 50.81)	2.263 (1.662 ~ 3.080) *	1.861 (1.328 ~ 2.609) *	2.303 (1.455 ~ 3.644) *
Blood selenium (ug/dl)			
Continuous log-transformed	0.760 (0.222 ~ 2.595)	0.961 (0.350 ~ 2.639)	1.083 (0.354 ~ 3.312)
Q1 (105.39 ~ 176.46)	Ref.		
Q2 (176.48 ~ 190.97)	1.020 (0.604 ~ 1.723)	1.090 (0.620 ~ 1.915)	1.010 (0.524 ~ 1.950)
Q3 (191.00 ~ 207.01)	0.986 (0.639 ~ 1.521)	1.104 (0.739 ~ 1.650)	0.998 (0.600 ~ 1.659)
Q4 (207.02 ~ 692.52)	0.905 (0.566 ~ 1.447)	0.978 (0.650 ~ 1.472)	1.016 (0.623 ~ 1.659)
Blood manganese (ug/dl)			
Continuous log-transformed	1.498 (1.001 ~ 2.240) *	1.287 (0.850 ~ 1.949)	1.317 (0.759 ~ 2.286)
Q1 (1.61 ~ 7.26)	Ref.		
Q2 (7.27 ~ 9.11)	1.093 (0.809 ~ 1.478)	1.124 (0.824 ~ 1.533)	1.106 (0.728 ~ 1.682)
Q3 (9.12 ~ 11.46)	1.233 (0.859 ~ 1.771)	1.234 (0.882 ~ 1.728)	1.092 (0.696 ~ 1.713)
Q4 (11.47 ~ 62.51)	1.500 (1.070 ~ 2.103)	1.262 (0.831 ~ 0.838)	1.268 (0.800 ~ 2.010)

Model 1: unadjusted; Model 2: adjusted for gender, age, race, education; Model 3: adjusted for gender, age, race, education, income, BMI, smoking, drinking. Including 4,054 participants in the analysis. *p < 0.05.Bold value means statistically significant.

Associations between blood metals and LTBI among adolescents are presented in Table 4, only blood manganese was positively associated with LTBI. The OR and 95% CIs for each one-unit increase in the log-transformed level of blood manganese with LTBI were 9.954 (1.389 ~ 71.344). Subgroup analyses of the association of blood manganese with LTBI showed significant associations in girls, aged ≥12 years and with a PIR > 3.0 (Supplementary Table S4).

**Table 4:** Associations between blood metals and LTBI among adolescents.

	Model 1	Model 2	Model 3
Blood lead (ug/dl)			
Continuous log-transformed	0.719 (0.158 ~ 3.278)	0.853 (0.245 ~ 2.963)	0.853 (0.221 ~ 3.297)
Q1 (0.18 ~ 0.45)	Ref.		
Q2 (0.46 ~ 0.62)	0.536 (0.148 ~ 1.937)	0.557 (0.172 ~ 1.808)	0.593 (0.192 ~ 1.830)
Q3 (0.63 ~ 0.90)	1.076 (0.186 ~ 6.216)	1.327 (0.320 ~ 5.503)	1.334 (0.304 ~ 5.860)
Q4 (0.91 ~ 15.37)	0.733 (0.114 ~ 4.709)	0.973 (0.211 ~ 4.492)	1.001 (0.192 ~ 5.227)
Blood cadmium (ug/dl)			
Continuous log-transformed	1.001 (0.274 ~ 3.663)	0.693 (0.131 ~ 3.675)	0.662 (0.129 ~ 3.410)
Q1 (0.11 ~ 0.11)	Ref.		
Q2 (0.16 ~ 0.19)	0.424 (0.102 ~ 1.762)	0.364 (0.076 ~ 1.751)	0.378 (0.079 ~ 1.810)
Q3 (0.20 ~ 0.21)	1.092 (0.267 ~ 4.461)	0.758 (0.140 ~ 4.098)	0.725 (0.137 ~ 3.839)
Total mercury (ug/dl)			
Continuous log-transformed	1.358 (0.854 ~ 2.160)	1.188 (0.664 ~ 2.125)	1.299 (0.656 ~ 2.570)
Q1 (0.11 ~ 0.22)	Ref.		
Q2 (0.23 ~ 0.37)	0.530 (0.046 ~ 6.075)	0.504 (0.041 ~ 6.161)	0.478 (0.038 ~ 6.018)
Q3 (0.38 ~ 0.68)	0.983 (0.222 ~ 4.342)	0.899 (0.187 ~ 4.316)	0.972 (0.205 ~ 4.602)
Q4 (0.69 ~ 9.39)	2.201 (0.423 ~ 11.456)	1.757 (0.247 ~ 12.475)	2.138 (0.261 ~ 17.497)
Blood selenium (ug/dl)			
Continuous log-transformed	8.005 (0.296 ~ 216.828)	2.631 (0.177 ~ 39.165)	2.747 (0.256 ~ 29.448)
Q1 (109.26 ~ 166.09)	Ref.		
Q2 (166.13 ~ 179.68)	1.216 (0.217 ~ 6.814)	1.124 (0.200 ~ 6.304)	1.068 (0.174 ~ 6.541)
Q3 (179.69 ~ 194.21)	2.036 (0.606 ~ 6.840)	1.730 (0.444 ~ 6.742)	1.712 (0.432 ~ 6.787)
Q4 (194.22 ~ 310.81)	1.767 (0.244 ~ 12.811)	1.223 (0.182 ~ 8.234)	1.240 (0.192 ~ 7.986)
Blood manganese (ug/dl)			
Continuous log-transformed	8.523 (1.893 ~ 38.368) *	8.298 (1.154 ~ 59.653) *	9.954 (1.389 ~ 71.344) *
Q1 (3.21 ~ 8.25)	Ref.		
Q2 (8.27 ~ 10.04)	12.730 (1.221 ~ 132.718)	12.781 (1.264 ~ 129.223)	11.905 (1.232 ~ 115.037)
Q3 (10.05 ~ 12.48)	12.578 (1.302 ~ 121.461)	13.229 (1.373 ~ 127.454)	13.977 (1.463 ~ 130.880)
Q4 (12.49 ~ 58.86)	31.576 (2.856 ~ 349.101)	31.666 (2.870 ~ 249.319)	32.292 (3.141 ~ 331.992)

Model 1: unadjusted; Model 2: adjusted for gender, age, race, education; Model 3: adjusted for gender, age, race, education and income. Including 1742 participants in the analysis. *p < 0.05.Bold value means statistically significant.

## Discussion

In the present study, we found positive relationships between total mercury and LTBI in US adults. Meanwhile, a positive association of blood manganese with LTBI among US adolescents was observed. The findings in the present study have important implications for public health, as both metal pollution and LTBI are major health concerns. The results indicated that controlling for environmental metals may be an effective measure to control LTBI. Besides, significant differences in age, educational levels, income and smoking status in participants with LTBI vs. non-LTBI in the present study were also observed.

The underlying biological mechanism between metals and LTBI remains unclear. The inflammation process is reported to be associated not only with heavy metals (21, 22), but also with LTBI (23). However, information on biomarkers of inflammation in NHANES data (2011 ~ 2012) is missing, and further study needs to be conducted in the future to explore the mechanism. In addition, heavy metals including mercury could increase lipid peroxidation and related oxidative stress (24, 25), and cardiometabolic risks (26, 27), which are also risk factors for LTBI (8, 28–30). However, these factors need to be confirmed in future studies involving animals and humans.

Subgroup analyses showed that the relationship between total mercury and LTBI was observed in US obese adults. A previous study showed that cumulative exposure to heavy metals was associated with obesity (31); meanwhile, obese individuals may be more susceptible to chronic systemic inflammation (32) and metabolic disorders (33), which may reinforce the risk of LTBI. On the other hand, their association was also observed in underweight adults, and a previous study showed that undernutrition was associated with gut microbiota composition and inflammation (34), which may provide some clue to understand the results; however, more studies are needed to explore the related mechanism. In addition, an association of total mercury was found among smokers, but not among nonsmokers. A previous study showed that the impact of smoking on heavy metal contamination (35, 36), and our study also revealed that the level of total mercury was higher in smokers than in nonsmokers. Similar findings were observed among drinkers, which may be due to the higher concentration of metals according to our results. Besides, the ORs of total mercury and LTBI in adults and blood manganese with LTBI in adolescents were different in sociodemographic characteristics, which implied more concerns should be given to certain population (29, 37, 38).

Our study was the first investigation to examine the relationship between metal exposure and LTBI among adults as well as in adolescents based on a large multiethnic, nationally representative sample of U.S. population. Our results suggested that more attention should be given to environmental pollution problems, especially for total mercury and blood manganese. However, some limitations should also be addressed. First, the data set is cross-sectional and causal inference could not be made. Second, our findings may be only representative of the US population, and further study should be conducted to validate the generalizability to other populations.

## Conclusion

The present study reveals that the positive associations between total mercury exposure and LTBI among adults and blood manganese are related to LTBI among adolescents in the United States. The findings indicate that exposure to specific metals is associated with increased prevalence of LTBI among adults and adolescents, which may have profound implications in light of the importance of controlling LTBI.

## Data availability statement

The original contributions presented in the study are included in the article/Supplementary material, further inquiries can be directed to the corresponding authors.

## Author contributions

JW: Software, Writing – original draft. KW: Formal analysis, Methodology, Software, Writing – original draft. FT: Investigation, Methodology, Writing – original draft. QL: Formal analysis, Writing – original draft. XL: Supervision, Writing – review & editing. FX: Conceptualization, Writing – review & editing.

## Footnotes

^1^ https://wwwn.cdc.gov/nchs/nhanes/analyticguidelines.aspx

^2^ https://wwwn.cdc.gov/Nchs/Nhanes/2011-2012/PBCD_G.htm
