# Supplementary material for: The association of blood metals with latent tuberculosis infection among adults and adolescents
Source: Front Nutr. 2023 Nov 3;10:1259902. doi: 10.3389/fnut.2023.1259902 (PMC10655142; doi:10.3389/fnut.2023.1259902)
Supplement: Supplementary file 1 [file Table_1.docx]

| **Table S1 Distribution of log-transformed blood metals in US adults (μg/dL; GM, 95%CI)** | | | | | | | | | |  |
| --- | --- | --- | --- | --- | --- | --- | --- | --- | --- | --- |
| **Characteristics** | **Blood lead** | ***P-value*** | **Blood cadmium** | ***P-value*** | **Total mercury** | ***P-value*** | **Blood selenium** | ***P-value*** | **Blood manganese** | ***P-value*** |
|  |  |  |  |  |  |  |  |  |  |  |
| **Gender** |  | <0.0001* |  | 0.1291 |  | 0.0425* |  | <0.0001* |  | <0.0001* |
| Male | 1.267 (1.184~1.356) |  | 0.300 (0.277~0.325) |  | 0.877 (0.765~1.005) |  | 195.888 (192.790~199.036) |  | 8.437 (8.271~8.606) |  |
| Female | 0.909 (0.858~0.963) |  | 0.364 (0.346~0.383) |  | 0.824 (0.718~0.946) |  | 190.161 (187.352~193.013) |  | 9.769 (9.574~9.969) |  |
| **Age** |  | <0.0001* |  | 0.0012* |  | 0.0031* |  | 0.0051* |  | <0.0001* |
| 18~44 | 0.778 (0.729~0.831) |  | 0.281 (0.261~0.303) |  | 0.709 (0.617~0.814) |  | 192.361 (190.008~194.742) |  | 9.418 (9.248~9.592) |  |
| 45~64 | 1.339 (1.241~1.444) |  | 0.374 (0.357~0.392) |  | 1.024 (0.882~1.188) |  | 195.291 (191.629~199.023) |  | 8.942 (8.649~9.244) |  |
| ≥65 | 1.599 (1.479~1.728) |  | 0.408 (0.377~0.443) |  | 0.949 (0.764~1.180) |  | 189.460 (184.592~194.456) |  | 8.583 (8.453~8.714) |  |
| **Race** |  | 0.0865 |  | <0.0001* |  | <0.0001* |  | 0.0026* |  | <0.0001* |
| Mexican American | 0.964 (0.873~1.065) |  | 0.272 (0.249~0.297) |  | 0.599 (0.532~0.674) |  | 193.156 (187.787~198.680) |  | 10.388 (9.999~10.793) |  |
| Non-Hispanic White | 1.085 (0.994~1.185) |  | 0.327 (0.304~0.353) |  | 0.819 (0.692~0.970) |  | 194.196 (190.230~198.245) |  | 8.853 (8.682~9.028) |  |
| Non-Hispanic Black | 1.061 (1.003~1.122) |  | 0.372 (0.350~0.396) |  | 0.825 (0.648~1.050) |  | 187.350 (183.601~191.176) |  | 8.248 (8.064~8.435) |  |
| Non-Hispanic Asian | 1.256 (1.170~1.349) |  | 0.464 (0.421~0.510) |  | 2.200 (1.828~2.648) |  | 196.172 (191.671~200.779) |  | 11.771 (11.325~12.236) |  |
| Others | 0.956 (0.859~1.063) |  | 0.316 (0.281~0.356) |  | 0.931 (0.819~1.059) |  | 188.607 (184.914~192.374) |  | 9.747 (9.343~10.168) |  |
| **Educational levels** |  | 0.0022* |  | 0.0013* |  | 0.0011* |  | 0.0248* |  | 0.0152* |
| Less than high school | 1.301 (1.200~1.410) |  | 0.418 (0.376~0.464) |  | 0.641 (0.556~0.739) |  | 188.832 (184.415~193.356) |  | 9.317 (8.934~9.715) |  |
| High school or equivalent | 1.126 (1.003~1.265) |  | 0.351 (0.322~0.383) |  | 0.707 (0.604~0.827) |  | 192.366 (187.048~197.834) |  | 8.831 (8.541~9.131) |  |
| College or above | 0.994 (0.941~1.050) |  | 0.306 (0.288~0.325) |  | 0.973 (0.832~1.137) |  | 194.197 (191.529~196.902) |  | 9.133 (8.962~9.308) |  |
| **PIR** |  | 0.1562 |  | 0.0007* |  | 0.0017* |  | 0.0147* |  | 0.021* |
| 0-1.0 | 1.028 (0.912~1.159) |  | 0.391 (0.328~0.466) |  | 0.627 (0.553~0.712) |  | 189.681 (187.072~192.326) |  | 9.362 (9.065~9.669) |  |
| 1.1-3.0 | 1.100 (0.997~1.214) |  | 0.356 (0.331~0.382) |  | 0.703 (0.627~0.789) |  | 191.197 (186.949~195.542) |  | 9.270 (9.064~9.481) |  |
| >3.0 | 1.041 (0.990~1.095) |  | 0.290 (0.270~0.310) |  | 1.087 (0.902~1.310) |  | 196.038 (192.627~199.510) |  | 8.909 (8.703~9.120) |  |
| **BMI, kg/m^2^** |  | 0.4532 |  | 0.0139* |  | 0.0015* |  | 0.1684 |  | 0.0433* |
| <25 | 1.095 (0.988~1.024) |  | 0.380 (0.335~0.430) |  | 0.927 (0.761~1.129) |  | 191.186 (188.707~193.698) |  | 9.074 (8.874~9.278) |  |
| 25-30 | 1.109 (1.036~1.188) | | 0.310 (0.292~0.329) |  | 0.936 (0.814~1.077) |  | 194.038 (190.446~197.699) |  | 8.946 (8.781~9.114) |  |
| ≥30 | 1.005 (0.941~1.074) |  | 0.312 (0.294~0.330) |  | 0.717 (0.649~0.793) |  | 193.648 (189.542~197.843) |  | 9.254 (9.025~9.487) |  |
| **Smoking status** |  | 0.0001* |  | <0.0001* |  | 0.077 |  | 0.9574 |  | 0.0019* |
| No | 0.942 (0.886~1.102) |  | 0.236 (0.225~0.248) |  | 0.879 (0.761~1.016) |  | 193.070 (190.077~196.110) |  | 9.343 (9.151~9.540) |  |
| Yes | 1.314 (1.216~1.420) |  | 0.529 (0.497~0.563) |  | 0.843 (0.718~0.990) |  | 192.890 (189.750~196.082) |  | 8.761 (8.512~9.017) |  |
| **Drinking status** |  | 0.0344* |  | 0.0026* |  | 0.1017 |  | 0.0011* |  | 0.0056* |
| No | 0.944 (0.880~1.012) |  | 0.314 (0.293~0.338) |  | 0.714 (0.627~0.813) |  | 188.982 (185.041~193.007) |  | 9.610 (9.184~10.055) |  |
| Yes | 1.104 (1.026~1.188) |  | 0.335 (0.317~0.355) |  | 0.873 (0.755~1.009) |  | 194.145 (191.153~197.185) |  | 8.946 (8.776~9.119) |  |

PIR: family income-poverty ratio; BMI: body mass index. * P<0.05.
