# Supplementary material for: The association of blood metals with latent tuberculosis infection among adults and adolescents
Source: Front Nutr. 2023 Nov 3;10:1259902. doi: 10.3389/fnut.2023.1259902 (PMC10655142; doi:10.3389/fnut.2023.1259902)
Supplement: Supplementary file 2 [file Table_2.docx]

| **Table S2 Distribution of log-transformed blood metals in US adolescents (μg/dL; GM, 95%CI)** | | | | | | | | | | |
| --- | --- | --- | --- | --- | --- | --- | --- | --- | --- | --- |
| **Characteristics** | **Blood lead** |  | **Blood cadmium** |  | **Total mercury** |  | **Blood selenium** |  | **Blood manganese** |  |
|  |  | ***P-value*** |  | ***P-value*** |  | ***P-value*** |  | ***P-value*** | **ug/dL (95%CI)** | ***P-value*** |
| **Gender** |  | <0.0001* |  | 0.8849 |  | 0.423 |  | 0.7583 |  | 0.0021* |
| Male | 0.675 (0.632~0.721) |  | 0.143 (0.138~0.149) |  | 0.348 (0.296~0.409) |  | 182.763 (178.186~187.457) |  | 9.770 (9.510~10.037) |  |
| Female | 0.541 (0.494~0.593) |  | 0.148 (0.142~0.156) |  | 0.362 (0.308~0.425) |  | 182.246 (177.048~187.597) |  | 10.696 (10.296~11.111) |  |
| **Age** |  | 0.0202* |  | <0.0001* |  | 0.0188* |  | <0.0001* |  | 0.6571 |
| 6~12 | 0.678 (0.622~0.740) |  | 0.131 (0.124~0.137) |  | 0.328 (0.285~0.378) |  | 176.025 (171.983~183.163) |  | 10.263 (9.976~10.558) |  |
| ≥12 | 0.548 (0.502~0.598) |  | 0.160 (0.153~0.167) |  | 0.378 (0.316~0.453) |  | 188.103 (182.348~194.040) |  | 10.196 (9.853~10.551) |  |
| **Race** |  | 0.0349* |  | 0.0012* |  | <0.0001* |  | 0.05 |  | <0.0001* |
| Mexican American | 0.563 (0.513~0.617) |  | 0.142 (0.133~0.151) |  | 0.339 (0.297~0.386） | | 184.877 (180.043~189.840) |  | 11.198 (10.837~11.571) |  |
| Non-Hispanic White | 0.573 (0.516~0.636) |  | 0.143 (0.136~0.150) |  | 0.308 (0.239~0.395) |  | 183.755 (176.615~191.184) |  | 10.210 (9.875~10.557) |  |
| Non-Hispanic Black | 0.723 (0.646~0.809) |  | 0.151 (0.144~0.158) |  | 0.416 (0.362~0.479) |  | 177.119 (173.810~180.490) |  | 8.493 (8.310~8.681) |  |
| Non-Hispanic Asian | 0.737 (0.627~0.868) |  | 0.183 (0.166~0.202) |  | 0.904 (0.759~1.076) |  | 181.485 (176.752~186.346) |  | 12.534 (11.562~13.608) |  |
| Others | 0.624 (0.537~0.725) |  | 0.147 (0.133~0.163) |  | 0.420 (0.358~0.492) |  | 180.919 (176.208~185.756) |  | 10.743 (10.163~11.357) |  |
| **PIR** |  | <0.0001* |  | 0.1904 |  | 0.0459* |  | 0.1149 |  | 0.8075 |
| 0-1.0 | 0.721 (0.672~0.773) |  | 0.144 (0.137~0.152) |  | 0.382 (0.340~0.427) |  | 179.502 (175.246~183.862) |  | 10.110 (9.766~10.467) |  |
| 1.1-3.0 | 0.594 (0.545~0.648) |  | 0.147 (0.140~0.155) |  | 0.309 (0.270~0.353) |  | 182.924 (177.885~188.105) |  | 10.221 (9.831~10.627) |  |
| >3.0 | 0.526 (0.484~0.572) |  | 0.144 (0.136~0.153) |  | 0.383 (0.286~0.514) |  | 183.516 (176.351~190.971) |  | 10.306 (9.790~10.848) |  |

PIR: family income-poverty ratio; BMI: body mass index. * P<0.05.
