# Supplementary material for: The association of blood metals with latent tuberculosis infection among adults and adolescents
Source: Front Nutr. 2023 Nov 3;10:1259902. doi: 10.3389/fnut.2023.1259902 (PMC10655142; doi:10.3389/fnut.2023.1259902)
Supplement: Supplementary file 3 [file Table_3.docx]

| **Table S3 Subgroup analysis of association between total mercury and LTBI among adults** | |
| --- | --- |
|  | OR (95%CI) |
| **Gender** |  |
| Male | 1.340 (1.011~1.775) |
| Female | 1.517 (1.009~2.279) |
| **Age** |  |
| 18~44 | 1.342 (1.003~1.796) |
| 45~64 | 1.457 (1.060~2.002) |
| ≥65 | 1.404 (1.117~1.765) |
| **Race** |  |
| Mexican American | 1.596 (1.070~2.380) |
| Non-Hispanic White | 1.773 (1.316~2.388) |
| Non-Hispanic Black | 1.222 (0.902~1.655) |
| Non-Hispanic Asian | 0.860 (0.654~1.130) |
| Others | 1.323 (1.043~1.678) |
| **Educational levels** |  |
| Less than high school | 1.093 (0.833~1.435) |
| High school or equivalent | 1.282 (0.943~1.743) |
| College or above | 1.645 (1.184~2.286) |
| **PIR** |  |
| 0-1.0 | 1.443 (0.978~2.129) |
| 1.1-3.0 | 1.206 (0.863~1.686) |
| >3.0 | 1.701 (1.217~2.376) |
| **BMI, kg/m^2^** |  |
| <25 | 1.380 (1.076~1.771) |
| 25-30 | 1.321 (0.889~1.964) |
| ≥30 | 1.553 (1.040~2.319) |
| **Smoking status** |  |
| No | 1.246 (0.913~1.701) |
| Yes | 1.535 (1.235~1.907) |
| **Drinking status** |  |
| No | 1.242 (0.802~1.924) |
| Yes | 1.464 (1.232~1.739) |

LTBI: latent tuberculosis infection; PIR: family income-poverty ratio; BMI: body mass index.

Adjusted for gender, age, race, education, income, BMI, smoking, drinking; log transformed total mercury is used in all analyses (n=4054).
