# Supplementary material for: The association of blood metals with latent tuberculosis infection among adults and adolescents
Source: Front Nutr. 2023 Nov 3;10:1259902. doi: 10.3389/fnut.2023.1259902 (PMC10655142; doi:10.3389/fnut.2023.1259902)
Supplement: Supplementary file 4 [file Table_4.docx]

| **Table S4 Subgroup analysis of association between blood manganese and LTBI among adolescents** | |
| --- | --- |
|  | OR (95%CI) |
| **Gender** |  |
| Boy | 2.857 (0.621~13.139) |
| Girl | 26.193 (1.257~546.007) |
| **Age** |  |
| 6~12 | 0.518 (0.148~1.811) |
| ≥12 | 45.576 (3.366~617.161) |
| **PIR** | |
| 0-1.0 | 6.731 (1.802~25.136) |
| 1.1-3.0 | 11.595 (0.790~170.134) |
| >3.0 | 12.479 (0.317~491.948) |

LTBI: latent tuberculosis infection; PIR: family income-poverty ratio.

Adjusted for gender, age, race, education and income; log transformed blood manganese is used in all analyses (n=1742)
