# Supplementary figures and images for: The association of blood metals with latent tuberculosis infection among adults and adolescents
Source: Front Nutr. 2023 Nov 3;10:1259902. doi: 10.3389/fnut.2023.1259902 (PMC10655142; doi:10.3389/fnut.2023.1259902)

**Figure S1 Flowchart of the study**

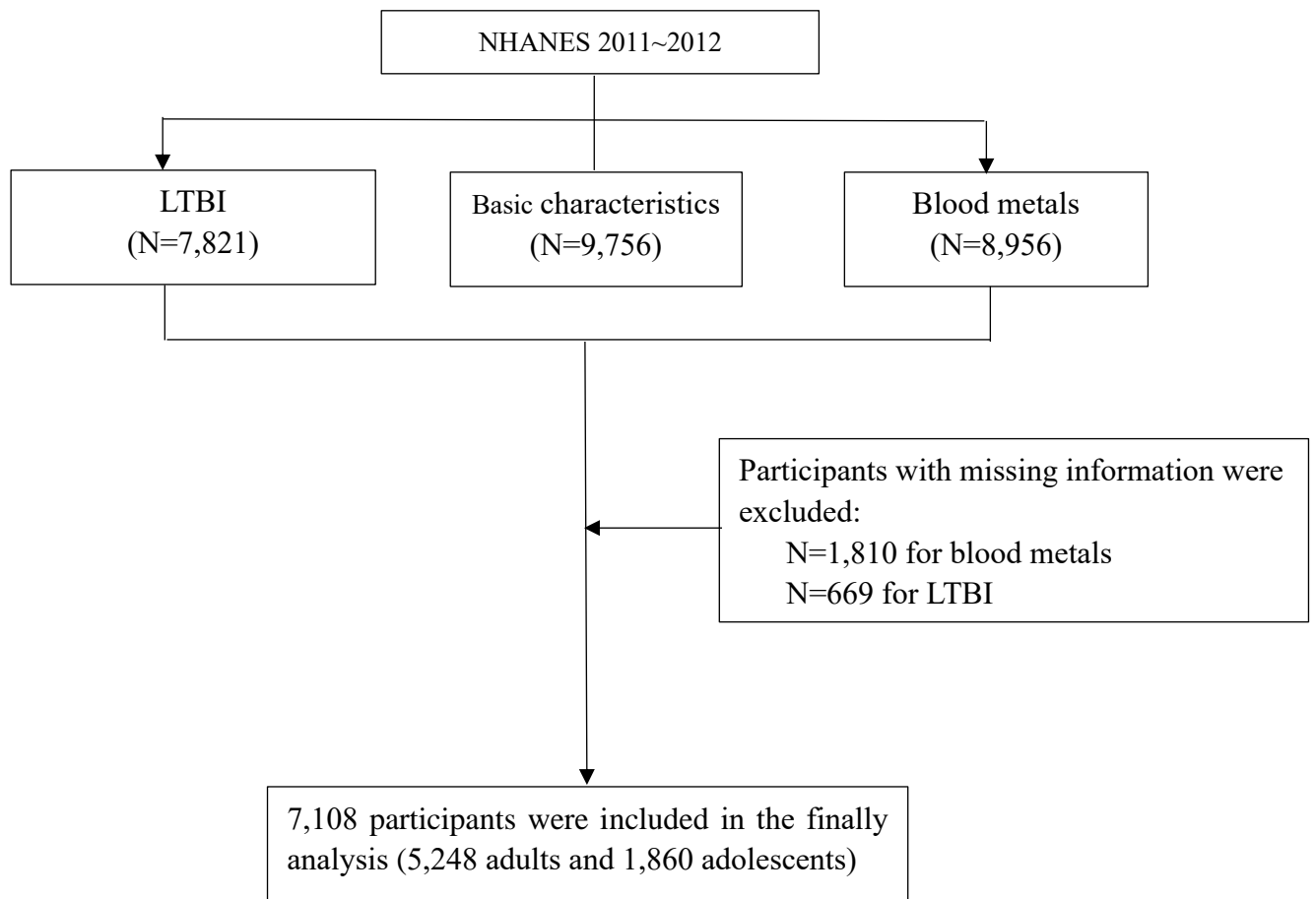

Supplement: Supplementary file 5 [file Image_1.pdf]
